# Supplementary material for: Mucus production, host-microbiome interactions, hormone sensitivity, and innate immune responses modeled in human cervix chips
Source: Nat Commun. 2024 May 29;15:4578. doi: 10.1038/s41467-024-48910-0 (PMC11137093; doi:10.1038/s41467-024-48910-0)
Supplement: Supplementary file 1 — Supplementary Information [file 41467_2024_48910_MOESM1_ESM.pdf]

SUPPLEMENTARY INFORMATION

Mucus production, host-microbiome interactions, hormone sensitivity, and innate immune responses modeled in human cervix chips

Zohreh Izadifar[1], Justin Cotton[1], Siyu Chen[2], Viktor Horvath[1], Anna Stejskalova[1], Aakanksha Gulati[1], Nina T. LoGrande[1], Bogdan Budnik[1], Sanjid Shahriar[1], Erin R. Doherty[1], Yixuan Xie[2], Tania To[1], Sarah E. Gilpin[1], Adama M. Sesay[1], Girija Goyal[1], Carlito Lebrilla[2], and Donald E. Ingber[1,3,4*]

[1]Wyss Institute for Biologically Inspired Engineering, Harvard University, Boston, MA 02215

[2]Department of Chemistry, University of California Davis, Davis, California, CA 95616

[3]Vascular Biology Program, Boston Children's Hospital and Department of Pathology, Harvard Medical School, Boston, MA 02115

[4]Harvard John A. Paulson School of Engineering and Applied Sciences, Cambridge, MA 02134

[*]Address all correspondence to: don.ingber@wyss.harvard.edu

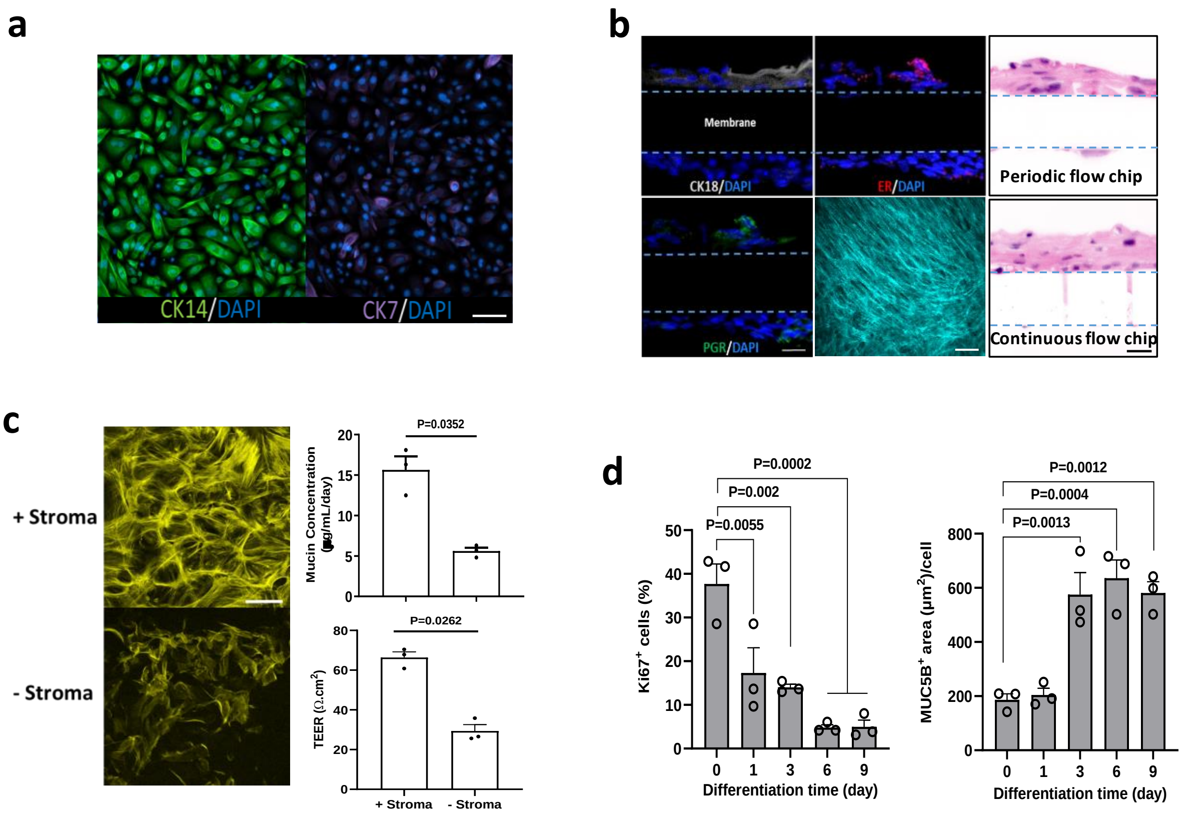

Supplementary Fig. S1. Characterization of the human Cervix Chip. a. Immunofluorescence micrographs of the cervical epithelial cells in 2D culture stained for cytokeratin 14 (CK14, green) (left) and cytokeratin 7 (CK7, magenta) (right) showing a mixture of primary human endo- and ecto-cervical epithelial cells, respectively (bar, 100 µm). **b.** Immunofluorescence microscopic vertical cross sectional views of the chip showing the cervical epithelium stained at day 7 of differentiation for cytokeratin 18 (CK18, white) (top left), estrogen receptor (ER, red) (middle top), progesterone receptor (PR, green) (bottom left), and Hoechst-stained nuclei (blue) (bar, 20 µm), and second harmonic generation microscopic planar view of the stroma on-chip showing an extensive network of long collagen fibers in the Cervix Chip (collagen, turquoise) (middle bottom) (bar, 50 µm). Pseudo-H&E cross section views of the Cervix Chips developed using periodic (top right) and continuous (bottom right) flow conditions. (bar, 20µm). **c.** Immunofluorescence microscopic views of cervical epithelium stained for F-actin (yellow) and viewed from above (bar 50 µm) (left) and graphs showing mucus levels (top right) and

transepithelial electrical resistance (TEER) (bottom right) in Transwell cervical epithelial cell cultures with (+) and without (-) stromal cells. **d.** Quantification of the change in the epithelial cells growth and differentiation in Cervix Chip measured by left) percentage change in the number of cells stained for Ki67, and right) surface area (μm^2) stained for MUC5B per cell in the epithelium throughout the culture time. Data represents the mean ± s.e.m.; n=3 (**c**), and 3 (**d**) experimental chip or Transwell replicates. Source data and statistical tests are provided as a Source Data file. Micrographs are representatives of three separate experiments.

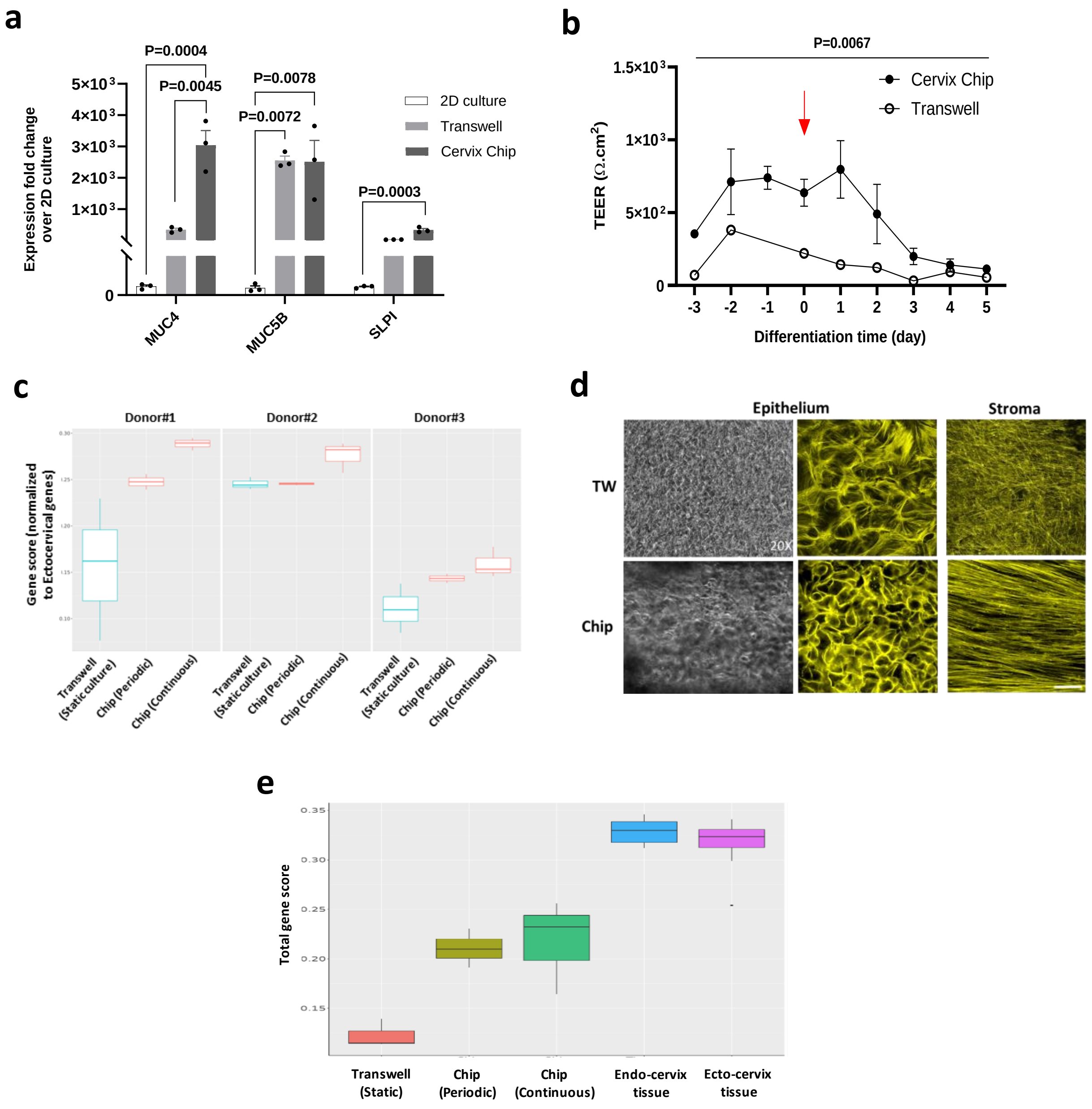

Supplementary Fig. S2. Cervix Chip comparison with static cultures. a. Graph showing fold change in expression of cervical epithelial genes encoding mucin 4 (*MUC4*), mucin 5B (*MUC5B*), and secretory leukocyte peptidase inhibition (*SLPI*) in periodic flow Cervix Chip and Transwell compared to 2D culture. **b.** Graph showing real-time changes in the Cervix Chip transepithelial electrical resistance TEER measured using custom made Organ Chip with integrated electrodes compared to Transwell culture measured using IVUM2 (red arrow indicates start of differentiation in culture). **c.** Gene set scoring

analysis showing relative ratio of the upregulated epithelial genes relative to the signature ectocervical genes in Cervix Chip cell developed using periodic and continuous flow regimen and Transwell culture for three different donors quantified using bulk RNAseq analysis of cervical epithelial cells. **d.** Left) Phase-contrast and (middle) immunofluorescence microscopic view of cervical epithelium in a Transwell (TW, top) and Cervix Chip (bottom) culture, and right) immunofluorescence microscopic view of fibroblast stroma stained for F-actin (yellow) (bar 50 µm). **e.** Gene set scoring analysis showing the relative ratio of the top 50% highly expressed collagens in GTEx tissue samples (*COL1A1, COL1A2, COL3A1, COL4A1, COL4A2, COL4A4, COL4A5, COL4A6, COL5A1, COL5A2, COL5A3, COL6A1, COL6A2, COL6A3, COL7A1, COL8A1, COL9A2, COL11A2, COL12A1, COL13A1, COL14A1, COL15A1, COL16A1, COL17A1, COL18A1, COL21A1, COL22A1, COL23A1, COL24A1, COL27A1*) in cervical epithelial cells of Cervix Chips developed using periodic and continuous flow regimen, and Transwell culture for three different donors, and 10 endocervical and 9 ectocervical tissue samples from GTEx quantified using bulk RNAseq analysis. Data represents the mean ± s.e.m.; n=3 (**a**), 8 (**b**), 3 (**c**), and 3 (**e**) experimental chip or Transwell replicates. Micrographs in (**d**) are representatives of three separate experiments. Source data and statistical tests are provided as a Source Data file.

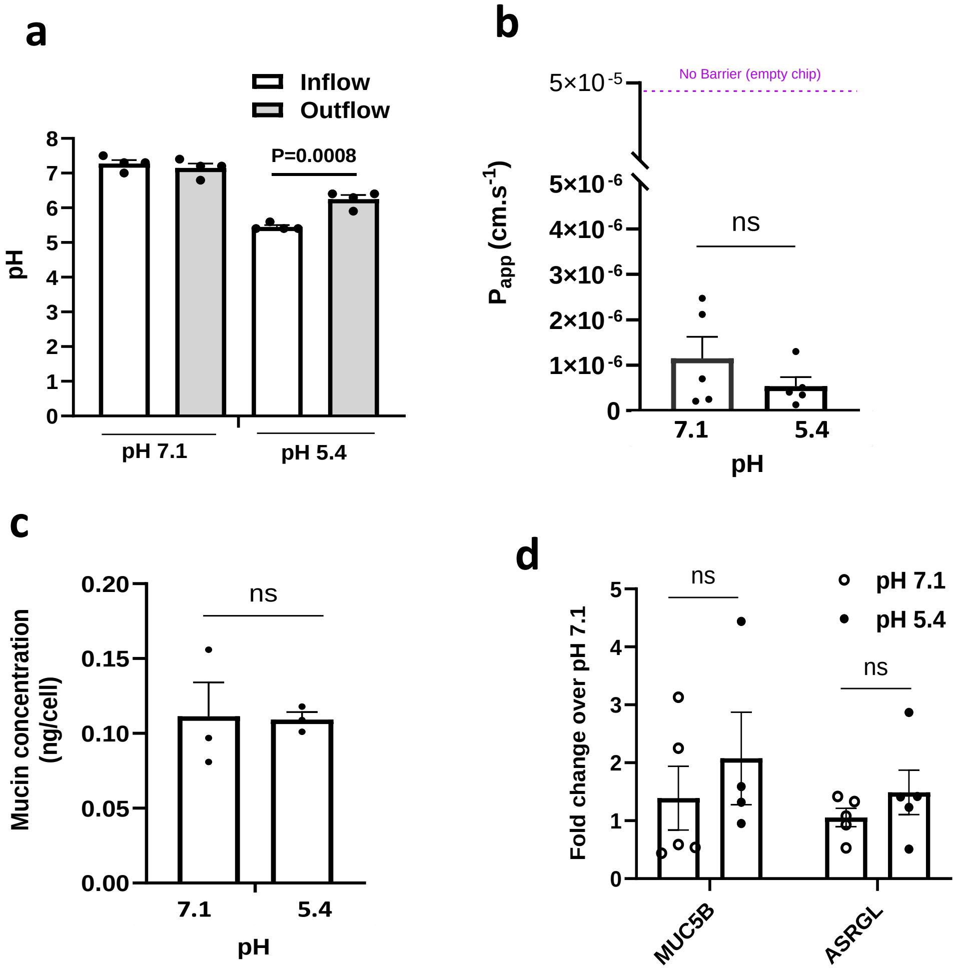

Supplementary Fig. S3. **Cervix Chip responses to changes in pH. a.** Graph showing changes in the pH of the Cervix Chip inflow and outflow media when cultured at pH 7.1 compared to pH 5.4. **b.** Quantification of permeability in Cervix Chip cultured under at pH 7.1 compared to pH 5.4 as measured by apparent permeability (P_{app}). **c.** Quantification of total mucin content produced by each cell in Cervix Chip cultured at pH 7.1 compared to pH 5.4 using an Alcian Blue assay. **d.** Graph showing fold change in the expression of cervical epithelial genes encoding mucin 5B (*MUC5B*) and asparaginase and isoaspartyl peptidase (*ASRGL*) in Cervix Chip cultured at pH 7.1 and pH 5.4 using real-time PCR. Data represent the mean ± s.e.m.; n=4 (**a**), 5 (**b**), 3 (**c**), and 5 (**d**) experimental chip replicates; *n.s.*, not significant Source data and statistical tests are provided as a Source Data file.

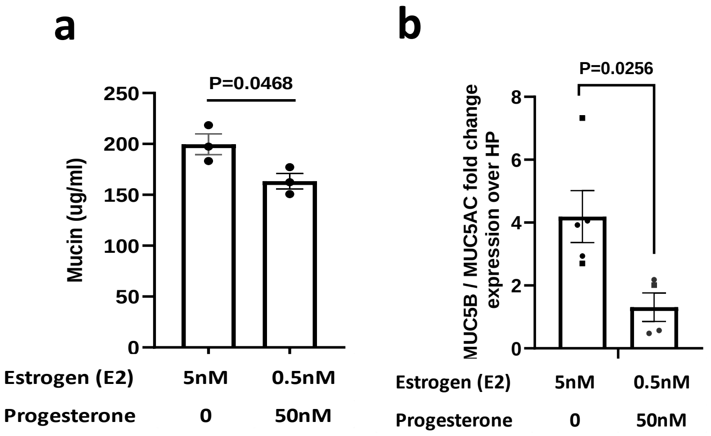

c

Class (Compound|Retention time)	Human Clinical Mucus	Cervix Chip HE	Cervix Chip HP
**O-glycans**			
Sialylated (2_2_0_2|28.2)	×	×	↓
Fucosylated (2_2_1_0|18.9)	×		
Sialylated and fucosylated (2_2_1_1|27.0)	×		
Sialylated (2_2_0_1|19.3)	×	×	↑
Sialylated and fucosylated (1_1_1_1|25.8)	×		
Fucosylated (2_2_2_0|21.9)	×		
Sialylated (3_3_0_1|21.6)	×		
Sialylated and fucosylated (2_2_1_2|24.7)	×	×	↓
Sialylated (3_3_0_2|24.8)	×	×	
Sialylated (2_2_0_2|26.1)	×		×
**N-glycans**			
Sialylated (6_5_0_3|30.9)	×	×	↓
Sialylated (5_4_0_2|25.9)	×	×	↓
Sialylated (5_4_0_2|25.1)	×	×	↓
Sialylated and fucosylated (5_4_1_2|26.7)	×	×	↓
Sialylated (5_4_0_1|24.0)	×	×	↓
Sialylated and fucosylated (6_5_1_3|29.3)	×		
Sialylated (7_6_0_3|31.5)	×		
Sialylated (5_4_0_2|27.5)	×	×	↓
Sialylated (6_5_0_2|26.6)	×		
Sialylated and fucosylated (5_4_1_1|25.3)	×	×	×
Sialylated (5_4_0_1|23.1)	×	×	↓
Fucosylated (5_4_1_0|22.6)	×	×	↓

Supplementary Fig. S4. Cervix Chip responses to sex hormones. a. Quantification of total mucin content produced by Cervix Chip cultured with high estrogen (5nM E2+0nM P4) compared to high progesterone (0.5nM E2+50nM P4) hormone levels using Alcian Blue assay. **b.** Graph showing change in expression level ratio of cervical genes encoding mucin 5B (*MUC5B*) and mucin 5AC (*MUC5AC*) in the Cervix Chips culture with high estrogen (5nM E2+0nM P4) compared to high progesterone (HP, 0.5nM E2+50nM P4) hormone levels. **c.** Changes in the abundance of O- and N-glycan compounds in the mucus collected from Cervix Chip cultured with high estrogen (HE, 5nM E2+0nM P4) compared to high progesterone (HP,

0.5nM E2+50nM P4) levels detected using nanoLC-QTOF MS/MS glycomic analysis (upward and downward arrows indicate increase and decrease, respectively, in the relative abundance of glycans in the HP compared to HE chips). Data represent the mean ± s.e.m.; n=3 (**a**), and 5 (**b**) experimental chip replicates. Source data and statistical tests are provided as a Source Data file.

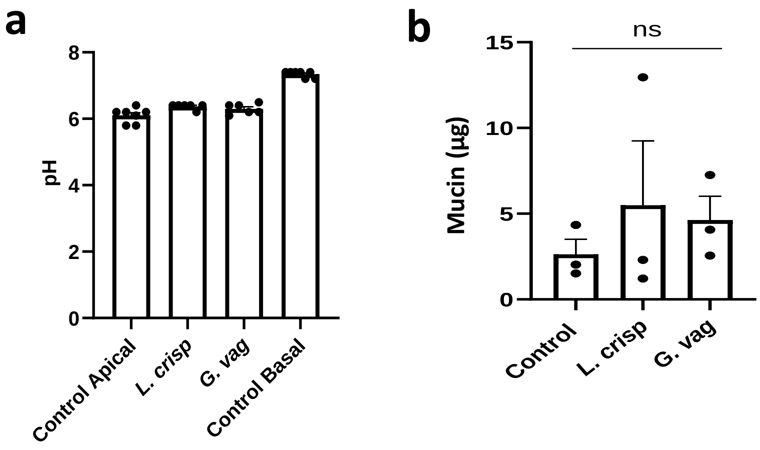

Supplementary Fig. S5. **Cervix Chip responses to bacteria. a.** Graph showing changes in the pH of the Cervix Chip epithelium effluents co-cultured in the absence or presence of *L. crispatus* or *G. vaginalis* consortia at 72 hours post inoculation compared to control basal channel. **b.** Quantification of total mucin content in the Cervix Chip effluents co-cultured with *L. crispatus* or *G. vaginalis* consortia compared to control chip without bacteria using an Alcian Blue assay. Data represent the mean ± s.e.m.; n=7 (**a**), and 3 (**b**) experimental chip replicates; n.s., not significant. Source data and statistical tests are provided as a Source Data file.
