# Supplementary material for: Mucus production, host-microbiome interactions, hormone sensitivity, and innate immune responses modeled in human cervix chips
Source: Nat Commun. 2024 May 29;15:4578. doi: 10.1038/s41467-024-48910-0 (PMC11137093; doi:10.1038/s41467-024-48910-0)
Supplement: Supplementary file 3 — Reporting Summary [file 41467_2024_48910_MOESM3_ESM.pdf]

nature portfolio

Corresponding author(s): Donald Ingber

Last updated by author(s): Dec 21, 2023

Reporting Summary

Nature Portfolio wishes to improve the reproducibility of the work that we publish. This form provides structure for consistency and transparency in reporting. For further information on Nature Portfolio policies, see our Editorial Policies and the Editorial Policy Checklist.

Statistics

For all statistical analyses, confirm that the following items are present in the figure legend, table legend, main text, or Methods section.

n/a	Confirmed	
☐	☒	The exact sample size (*n*) for each experimental group/condition, given as a discrete number and unit of measurement
☐	☒	A statement on whether measurements were taken from distinct samples or whether the same sample was measured repeatedly
☐	☒	The statistical test(s) used AND whether they are one- or two-sided<br>*Only common tests should be described solely by name; describe more complex techniques in the Methods section.*
☒	☐	A description of all covariates tested
☐	☒	A description of any assumptions or corrections, such as tests of normality and adjustment for multiple comparisons
☐	☒	A full description of the statistical parameters including central tendency (e.g. means) or other basic estimates (e.g. regression coefficient) AND variation (e.g. standard deviation) or associated estimates of uncertainty (e.g. confidence intervals)
☐	☒	For null hypothesis testing, the test statistic (e.g. *F*, *t*, *r*) with confidence intervals, effect sizes, degrees of freedom and *P* value noted<br>*Give P values as exact values whenever suitable.*
☒	☐	For Bayesian analysis, information on the choice of priors and Markov chain Monte Carlo settings
☒	☐	For hierarchical and complex designs, identification of the appropriate level for tests and full reporting of outcomes
☒	☐	Estimates of effect sizes (e.g. Cohen's *d*, Pearson's *r*), indicating how they were calculated

Our web collection on statistics for biologists contains articles on many of the points above.

Software and code

Policy information about availability of computer code

Data collection	CompactStat.h Mobile electrochemical interface (B32121, Ivium Technologies B. V., The Netherlands), Epithelial Voltohmmeter and EndOhm Culture Cup Chamber (WPI, USA), Leica microscopy Software (SP5 X MP DMI-6000), IviumSoft (V4.97, Informer Technologies, Inc.), BioTek microplate reader (BioTeK NEO, Gen5 3.11), microplate reader (Syn-Q55 Synergy HT, BioTek), Inverted microscope (Axial observer Z1 Zeiss, OCRA-Flash4.0 C11440 Hamamatsu camera), Zeiss Zen microscopy Software (BLUE version), Revolver microscopy Software (V6.0.1), MSD Discovery Workbench machine (Model 1300) and Software (4.0.12), Agilent 1200 series HPLC-Chip (PGC), Agilent 6520 Accurate-Mass Q-TOF MS (Agilent, CA), HFX Orbitrap (ThermoScientific, Germany) equipped with dual 3000 nano-HPLC pump (ThermoScientific, Germany).
Data analysis	Imaris software (Bitplaneversion V9.3), GraphPad Prism (V 8.1.2, 2019), Fiji (V1.8), STAR (v2.5.2b) , Subread package (v2.0.3), R (v3.6.3), DESeq2 (v.1.26.0), and ggplot2 (v3.3.5), heatmap (v1.0.12), singscore (v1.6.0), Matlab (R2021a), Agilent MassHunter Qualitative Analysis Software(B.08), Proteome Discoverer 2.5 (Thermo Scientific, CA) software.

For manuscripts utilizing custom algorithms or software that are central to the research but not yet described in published literature, software must be made available to editors and reviewers. We strongly encourage code deposition in a community repository (e.g. GitHub). See the Nature Portfolio guidelines for submitting code & software for further information.

Data

Policy information about availability of data

All manuscripts must include a data availability statement. This statement should provide the following information, where applicable:

- Accession codes, unique identifiers, or web links for publicly available datasets
- A description of any restrictions on data availability
- For clinical datasets or third party data, please ensure that the statement adheres to our policy

The sequencing data reported in this paper have been deposited in the GEO database under accession no. GSE231016 [https://www.ncbi.nlm.nih.gov/geo/query/acc.cgi?acc=GSE231016]. Gene expression profiles from human endocervix and ectocervix were obtained from GTEx analysis V8 data accessed on GTEx Portal (https://www.gtexportal.org, accession no. GSE168244) on 01/05/2022. The raw glycomic data reported in this paper can be found on the MassIVE repository [http://doi:10.25345/C52805830, accession no. MSV000091806). The raw proteomic data generated in this study are deposited at MassIVE repository [https://doi.org/doi:10.25345/C5F18SR7P, accession no. MSV000093636]. The used human and bacteria protein sequence databases are accessible at Uniprot database [human in UniProtKB search (51829) | UniProt and bacteria in UniProtKB search (341308) | UniProt]. All other data reported in this study are available within the paper, its Supplementary Information or Source Data files provided with this paper.

Research involving human participants, their data, or biological material

Policy information about studies with human participants or human data. See also policy information about sex, gender (identity/presentation), and sexual orientation and race, ethnicity and racism.

Reporting on sex and gender	Primary human cervical epithelial cells (female source) were purchased from commercial vendors. Primary human cervical fibroblast cells were isolated from healthy cervical tissues removed from female patients underwent hysterectomy procedure under Massachusetts General Hospital Institutional Review Board-approved protocol #2015P001859.
Reporting on race, ethnicity, or other socially relevant groupings	The ethnicity of the human cervical epithelial cells reported in this manuscript were based on the identified and reported ethnicity by the commercial vendor where the primary cervical epithelial cells were purchased from (LifeLine Cell Technology).
Population characteristics	One human patient was used in this study for isolation of primary stromal cells. The patient was a 37 years old female (at-birth assigned gender) that went under hysterectomy procedure for uterine fibroids.
Recruitment	The human research participant was selected in accordance with the method described in the Institutional Review Board of Mass General Brigham (Protocol number 2015P001859). Informed consent was not obtained because the samples were deidentified. No compensation was provided to the participants.
Ethics oversight	All methods were carried out in accordance with the approval of the Institutional Review Board of Wyss Institute for Biologically Inspired Engineering at Harvard University (Protocol number IRB22632) and Mass General Brigham (Protocol number 2015P001859).

Note that full information on the approval of the study protocol must also be provided in the manuscript.

Field-specific reporting

Please select the one below that is the best fit for your research. If you are not sure, read the appropriate sections before making your selection.

☒ Life sciences ☐ Behavioural & social sciences ☐ Ecological, evolutionary & environmental sciences

For a reference copy of the document with all sections, see nature.com/documents/nr-reporting-summary-flat.pdf

Life sciences study design

All studies must disclose on these points even when the disclosure is negative.

Sample size	For chip experiments, at least 3-6 chips were used based on prior reports on organ chip variations as previously described (Mahajan et al., Microbiome, 2022, Si et al., Nature Biomedical Engineering, 2021; Bein et al., Nature Biomedical Engineering, 2021). In all other experiments , at least 3 biological replicates, and two independent experiments were included. The exact number of sample size for each experiment is indicated in the figure legends. Statistical methods were not performed for predetermination of sample size.
Data exclusions	Data from Organ Chips that exhibited signs of poor cell viability and tissue differentiation prior to hormonal or bacterial treatment were excluded from analysis. Sensor chips that showed failure from fabrication imperfections (inconsistent background TEER measurements, leaks) during experiment were excluded from data collection and analysis.
Replication	In order to verify the reproducibility of experimental findings, all Organ Chip experiments were performed at least in triplicate. All attempts at replication were successful.
Randomization	All Organ Chips were randomly allocated into groups.

Blinding

Data collection and analysis for cytokines, glycomics, proteomics, RT-qPCR, and RNA-seq were run blinded since sample collection, and data extraction and analysis were performed by different researchers (internally or through external collaborations) or third party (Azenta Life Sciences) that could not identify the samples based on the assigned labels. Blinding was not applicable to live imaging and other quantitative data collections during experiments, since the experiments were controlled.

Reporting for specific materials, systems and methods

We require information from authors about some types of materials, experimental systems and methods used in many studies. Here, indicate whether each material, system or method listed is relevant to your study. If you are not sure if a list item applies to your research, read the appropriate section before selecting a response.

Materials & experimental systems

n/a	Involved in the study	
☐	☒	Antibodies
☐	☒	Eukaryotic cell lines
☒	☐	Palaeontology and archaeology
☒	☐	Animals and other organisms
☒	☐	Clinical data
☒	☐	Dual use research of concern
☒	☐	Plants

Methods

n/a	Involved in the study	
☒	☐	ChIP-seq
☒	☐	Flow cytometry
☒	☐	MRI-based neuroimaging

Antibodies

Antibodies used

Primary Antibodies:
Hoescht 33342 (Life Technologies, H3570, dilution 1:1000), MUC5B (Abcam, Cat. no. ab87376, dilution 1:200), KI67 (Thermo Scientific, Cat. no. RM-9106-S, dilution 1:200), cytokeratin 18 (Abcam, Cat. no. ab668, dilution 1:500), cytokeratin 7 (Abcam, Cat. No. ab209601, dilution 1:100), cytokeratin 14 (Abcam, Cat. No. ab51054, dilution 1:200), F-actin (Abcam, Cat. no. ab176757, dilution 1:200), vimentin (Abcam, Cat. No. 195878, dilution 1:1000), estrogen receptor (Abcam, Cat. no. ab32063, dilution 1:200), and progesterone receptor (Abcam, Cat. no. ab2765, dilution 1:200), Eosin Y solution (Abcam, cat. no. ab246824), no dilution), DAPI (Invitrogen, cat. no. D1306, dilution 1:1000).
Secondary antibodies:
Donkey anti-rabbit Alexa Flour 647 (Jackson lab, Cat. no. 715-605-151, dilution 1:500 and 715-605-152, dilution 1:500) and Donkey anti-rabbit Alexa Flour 488 (ThermoFisher, Cat. no. A21206, dilution 1:500).

Validation

All antibodies validation data are available on the manufacturer's website, and we verified this information with the provided Data Sheets from the manufactures. We also confirmed the specificity of antibodies for human immunofluorescence staining by validating and optimizing the antibodies dilutions with human cervical tissue samples.

Eukaryotic cell lines

Policy information about cell lines and Sex and Gender in Research

Cell line source(s)

Primary cervical epithelial (CE) cells (LifeLine Cell Technology Cat# FC-0080, Donors ID: 6225 (African American), 6226 (Hispanic), 6227(Caucasian). Primary cervical fibroblast (healthy cervical tissue obtained from hysterectomy procedure, Massachusetts General Hospital)

Authentication

All cells showed expected cell morphology, growth behavior, and were confirmed with positive expression of known markers for human cervical epithelial and stromal fibroblast cells.

Mycoplasma contamination

Cervical epithelial cells were tested for endotoxin and mycoplasma by the vendors.

Commonly misidentified lines
(See ICLAC register)

No commonly misidentified cell lines were used.
